# SAT Solving with GPU Accelerated Inprocessing

**DOI:** 10.1007/978-3-030-72016-2_8

**Published:** 2021-03-01

**Authors:** Muhammad Osama, Anton Wijs, Armin Biere

**Affiliations:** 8 Eindhoven University of Technology, Eindhoven, The Netherlands; 9 Aalborg University, Aalborg East, Denmark; 10 Eindhoven University of Technology, Eindhoven, The Netherlands; 11 Johannes Kepler University, Linz, Austria

**Keywords:** Satisfiability, Variable Elimination, Eager Redundancy Elimination, Parallel SAT Inprocessing, Parallel Garbage Collection, GPU

## Abstract

Since 2013, the leading SAT solvers in the SAT competition all use inprocessing, which unlike preprocessing, interleaves search with simplifications. However, applying inprocessing frequently can still be a bottle neck, i.e., for hard or large formulas. In this work, we introduce the first attempt to parallelize inprocessing on GPU architectures. As memory is a scarce resource in GPUs, we present new space-efficient data structures and devise a data-parallel garbage collector. It runs in parallel on the GPU to reduce memory consumption and improves memory access locality. Our new parallel variable elimination algorithm is twice as fast as previous work. In experiments our new solver ParaFROST solves many benchmarks faster on the GPU than its sequential counterparts.

### 1 Introduction

During the past decade, SAT solving has been used extensively in many applications, such as combinational equivalence checking [27], automatic test pattern generation [33, 40], automatic theorem proving [14], and symbolic model checking [7, 13]. Simplifying SAT problems prior to solving them has proven its effectiveness in modern conflict-driven clause learning (CDCL) SAT solvers [5, 6, 17], particularly when applied on real-world applications relevant to software and hardware verification [16, 20, 22, 24].

Since 2013, simplification techniques [8, 16, 19, 21, 41] are also used periodically *during* SAT solving, which is known as *inprocessing* [3–6, 23]. Applying inprocessing iteratively to large problems can be a performance bottleneck in SAT solving procedure, or even increase the size of the formula, negatively impacting the solving time.

Graphics processors (GPUs) have become attractive for general-purpose computing with the availability of the Compute Unified Device Architecture (CUDA) programming model. CUDA is widely used to accelerate applications that are computationally intensive w.r.t. data processing. For instance, we have applied GPUs to accelerate explicit-state model checking [11, 43], bisimilarity checking [42], the reconstruction of

* This work is part of the GEARS project with project number TOP2.16.044, which is (partly) financed by the Netherlands Organisation for Scientific Research (NWO).
† We gratefully acknowledge the support of NVIDIA Corporation with the donation of the GeForce Titan RTX used for this research.
‡ Partially funded by the LIT AI Lab.

genetic networks [12], wind turbine emulation [30], metaheuristic SAT solving [44], and SAT-based test generation [33]. Recently, we introduced SIGmA [34, 35] as the first SAT simplification preprocessor to exploit GPUs.

**Contributions.** Embedding GPU inprocessing in a SAT solver is highly non-trivial and has never been attempted before, according to the best of our knowledge. Efficient data structures are needed that allow parallel processing, and that support efficient adding and removing of clauses. For this purpose, we contribute the following:

1. We propose a new dynamically expanded data structure for clauses supporting both 32-bit [17] and 64-bit references with a minimum of 20 bytes per clause.
2. A new parallel garbage collector is presented, tailored for GPU inprocessing.
3. Our new parallel variable elimination algorithm is twice as fast as [34] and together with other improvements yields much higher performance and robustness.
4. Our parallel inprocessing is deterministic (i.e., results are reproducible).

In addition, we propose a new preprocessing technique targeted towards data-parallel execution, called *Eager Redundancy Elimination* (ERE), which is applicable on both original and learnt clauses. All contributions have been implemented in our solver ParaFROST and benchmarked on a larger set than considered previously in [34], using 493 application problems. We discuss the potential performance gain of the GPU inprocessing and its impact on SAT solving, compared to a sequential version of our solver as well as CaDiCaL [6], a state-of-the-art solver developed by the last author.

### 2 Preliminaries

All SAT formulas in this paper are in conjunctive normal form (CNF). A CNF formula is a conjunction of $m$ clauses $\bigwedge_{i=1}^{m} C_i$, where each clause $C_i$ is a disjunction of $k$ literals $\bigvee_{j=1}^{k} \ell_j$, and a literal is a Boolean variable $x$ or its complement $\neg x$, which we refer to as $\bar{x}$. We represent clauses by sets of literals, i.e., $\{\ell_1, \ldots, \ell_k\}$ represents the formula $\ell_1 \vee \ldots \vee \ell_k$, and a SAT formula by a set of clauses, i.e., $\{C_1, \ldots, C_m\}$ represents the formula $C_1 \wedge \ldots \wedge C_m$. With $\mathcal{S}_\ell$, we refer to the set of clauses containing literal $\ell$, i.e., $\mathcal{S}_\ell = \{C \in \mathcal{S} \mid \ell \in C\}$. If for a variable $x$, we have either $\mathcal{S}_x = \emptyset$ or $\mathcal{S}_{\bar{x}} = \emptyset$ (but not both), then the literal $\bar{x}$ or $x$, respectively, is called a *pure literal*. A clause $C$ is a *tautology* iff there exists a variable $x$ with $\{x, \bar{x}\} \subseteq C$, and $C$ is *unit* iff $|C| = 1$.

In this paper we integrate GPU-accelerated inprocessing and CDCL [28, 32, 36]. One important aspect of CDCL is to learn from previous assignments to prune the search space and make better decisions in the future. This learning process involves the periodic adding of new *learnt* clauses to the input formula while CDCL is running.

In this paper, clauses are either considered to be `LEARNT` or `ORIGINAL` (*redundant* and *irredundant* in [23] and in the SAT solver CaDiCaL [6]). A `LEARNT` clause is added to the formula by the CDCL clause learning process, and an `ORIGINAL` clause is part of the formula from the very start. Furthermore, each assignment is associated with a *decision level* that acts as a time stamp, to monitor the order in which assignments are performed. The first assignment is made at decision level one.

**Variable Elimination (VE).** Variables can be removed from clauses by either applying the *resolution rule* or *substitution* (also known as gate equivalence reasoning) [16, 23].

Concerning the former, we represent application of the resolution rule w.r.t. some variable $x$ using a *resolving operator* $\otimes_x$ on clauses $C_1$ and $C_2$. The result of applying the rule is called the *resolvent* [41]. It is defined as $C_1 \otimes_x C_2 = C_1 \cup C_2 \setminus \{x, \bar{x}\}$, and can be applied iff $x \in C_1$, $\bar{x} \in C_2$. The $\otimes_x$ operator can be extended to resolve sets of clauses w.r.t. variable $x$. For a formula $\mathcal{S}$, let $\mathcal{L} \subset \mathcal{S}$ be the set of learnt clauses when we apply the resolution rule. The set of new resolvents is then defined as $R_x(\mathcal{S}) = \{C_1 \otimes_x C_2 \mid C_1 \in S_x \setminus \mathcal{L} \wedge C_2 \in S_{\bar{x}} \setminus \mathcal{L} \wedge \neg\exists y.\{y, \bar{y}\} \subseteq C_1 \otimes_x C_2\}$. Notice that the learnt clauses can be ignored [23] (i.e., in practice, it is not effective to apply resolution on learnt clauses). The last condition avoids that a resolvent should not be a tautology. After eliminating variable $x$ in $\mathcal{S}$, the resulting formula $\mathcal{S}'$ is defined as $\mathcal{S}' = R_x(\mathcal{S}) \cup (\mathcal{S} \setminus (\mathcal{S}_x \cup \mathcal{S}_{\bar{x}}))$, i.e., the new resolvents are combined with the original and learnt clauses that do not reference $x$.

Substitution detects patterns encoding logical gates, and substitutes the involved variables with their gate-equivalent counterparts. Previously [34], we only considered AND gates. In the current work, we add support for *Inverter*, *If-Then-Else* and *XOR* gate extractions. For all logical gates, substitution can be performed by resolving non-gate clauses (i.e., clauses not contributing to the gate itself) with gate clauses [23].

For instance, the first three clauses in the formula $\{\{x, \bar{a}, \bar{b}\}, \{\bar{x}, a\}, \{\bar{x}, b\}, \{x, c\}\}$ together encode a logical AND-gate, hence the final clause can be resolved with the second and the third clauses, producing the simplified formula $\{\{a, c\}, \{b, c\}\}$. Combining gate equivalence reasoning with the resolution rule tends to result in smaller formulas compared to only applying the resolution rule [16, 23, 37].

**Subsumption elimination.** SUB performs *self-subsuming resolution* followed by *subsumption elimination* [16]. The former can be applied on clauses $C_1, C_2$ iff for some variable $x$, we have $C_1 = C_1' \cup \{x\}$, $C_2 = C_2' \cup \{\bar{x}\}$, and $C_2' \subseteq C_1'$. In that case, $x$ can be removed from $C_1$. The latter is applied on clauses $C_1, C_2$ with $C_2 \subseteq C_1$. In that case, $C_1$ is redundant and can be removed. If $C_2$ is a `LEARNT` clause, it must be considered as `ORIGINAL` in the future, to prevent deleting it during learnt clause reduction, a procedure which attempts to reduce the number of learnt clauses [6, 23]. For instance, consider the formula $\mathcal{S} = \{\{a, b, c\}, \{\bar{a}, b\}, \{b, c, d\}\}$. The first clause is self-subsumed by the second clause w.r.t. variable $a$ and can be strengthened to $\{b, c\}$ which in turn subsumes the last clause $\{b, c, d\}$. The latter clause is then removed from $\mathcal{S}$ and the simplified formula becomes $\{\{b, c\}, \{\bar{a}, b\}\}$.

**Blocked clause elimination.** BCE [25] can remove clauses for which variable elimination always results in tautologies. Consider the formula $\{\{a, b, c\}, \{\bar{a}, \bar{b}\}, \{\bar{a}, \bar{c}\}\}$. All three literals $a$, $b$ and $c$ are blocking the first clause, since resolving $a$ produces the tautologies $\{\{b, c, \bar{b}\}, \{b, c, \bar{c}\}\}$, resolving $b$ produces $\{\bar{a}, a, c\}$, and resolving $c$ produces $\{\bar{a}, a, b\}$. Hence the blocked clause $\{a, b, c\}$ can be removed from $\mathcal{S}$. Again, as for VE, only original clauses are considered.

**Eager Redundancy Elimination.** ERE is a new elimination technique that we propose, which repeats the following until a fixpoint has been reached: for a given formula $\mathcal{S}$ and clauses $C_1 \in \mathcal{S}, C_2 \in \mathcal{S}$ with $x \in C_1$ and $\bar{x} \in C_2$ for some variable $x$, if there exists a clause $C \in \mathcal{S}$ for which $C \equiv C_1 \otimes_x C_2$, then let $\mathcal{S} := \mathcal{S} \setminus \{C\}$. In this work, we restrict removing $C$ to the condition ($C_1$ is `LEARNT` $\vee$ $C_2$ is `LEARNT`) $\implies$ $C$ is `LEARNT`.

If the condition holds, $C$ is called a *redundancy* and can be removed without altering the original satisfiability. For example, consider $\mathcal{S} = \{\{a, \bar{c}\}, \{c, b\}, \{\bar{d}, \bar{c}\}, \{b, a\}, \{a, d\}\}$. Resolving the first two clauses gives the resolvent $\{a, b\}$ which is equivalent to the fourth clause in $\mathcal{S}$. Also, resolving the third clause with the last clause yields $\{a, \bar{c}\}$ which is equivalent to the first clause in $\mathcal{S}$. ERE can remove either $\{a, \bar{c}\}$ or $\{a, b\}$ but not both. Note that this method is entirely different from *Asymmetric Tautology Elimination* in [21]. The latter requires adding so-called hidden literals to all clauses to check which is a hidden tautology. ERE can operate on learnt clauses and does not require literals addition, making it more effective and adequate to data parallelism.

### 3 GPU Memory and Data Structures

**GPU Architecture.** Since 2007, NVIDIA has been developing a parallel computing platform called CUDA [31] that allows developers to use GPU resources for general purpose processing. A GPU contains multiple streaming multiprocessors (SMs), each SM consisting of an array of streaming processors (SPs). Every SM can execute multiple threads grouped together in 32-thread scheduling units called *warps*.

A GPU computation can be launched in a program by the *host* (CPU side of a program) by calling a GPU function called a *kernel*, which is executed by the *device* (GPU side of a program). When a kernel is called, it is specified how many threads need to execute it. These threads are partitioned into thread *blocks* of up to 1,024 threads (or 32 warps). Each block is assigned to an SM. All threads together form a *grid*. A hardware warp scheduler evenly distributes the launched blocks to the available SMs. Concerning the memory hierarchy, a GPU has multiple types of memory:

- *Global memory* with high bandwidth but also high latency is accessible by both GPU threads and CPU threads and thus acts as interface between CPU and GPU.
- *Constant memory* is read-only for all GPU threads. It has a lower latency than global memory, and can be used to store any pre-defined constants.
- *Shared memory* is on-chip memory shared by the threads in a block. Each SM has its own shared memory. It is much smaller in size than global and constant memory (in the order of tens of kilobytes), but has a much lower latency. It can be used to efficiently communicate data between threads in a block.
- *Registers* are used for on-chip storage of thread-local data. It is very small, but provides the fastest memory.

To hide the latency of global memory, ensuring that the threads perform *coalesced accesses* is one of the best practices. When the threads in a warp try to access a consecutive block of 32-bit words, their accesses are combined into a single (coalesced) memory access. Uncoalesced memory accesses can, for instance, be caused by data sparsity or misalignment. Furthermore, we use *unified memory* [31] to store the main data structures that need to be regularly accessed by both the CPU and the GPU. Unified memory creates a pool of managed memory that is shared between the CPU and GPU. This pool is accessible to both sides using the same addresses. Regarding atomicity, a GPU can run *atomic* instructions on both global and shared memory. Such an instruction performs a *read-modify-write* memory operation on one 32-bit or 64-bit word.

```
class SCLAUSE {
    char state, flag;
    char added, used;
    int size, lbd;
    uint32 sig;
    union {
        uint32 literals[1];
    };
}
```

(a) container for a clause

```
class CNF {
    struct {
        uint32* memory;
        uint64 size, cap;
    } clauses;
    struct {
        uint64* memory;
        uint32 size, cap;
    } references;
}
```

(b) container for a formula

Fig. 1: Data structures to store a SAT formula on a GPU

**Data Structures.** To efficiently implement inprocessing techniques for GPU architectures, we designed a new data structure from scratch to count the number of learnt clauses, and store other relevant clause information, while keeping the memory consumption as low as possible. Fig. 1 shows the proposed structures to store a clause (denoted by `SCLAUSE`) and the SAT formula represented in CNF form (denoted by `CNF`). The `state` member in Fig. 1a stores the current *clause state*. A clause is either `ORIGINAL`, `LEARNT` (see Section 2) or `DELETED`. A GPU thread is not allowed to deallocate memory, however, a clause can be set to `DELETED` and freed later during garbage collection. The members `added` and `flag` mark the clause for being resolvent (when applying the resolution rule) and contributing to a gate (for substitution), respectively. The `lbd` entry denotes the *literal block distance* (LBD), i.e., the number of decision levels contributing to a conflict [2]. The `used` counter is used to keep track of how long a `LEARNT` clause should be used before it gets deleted during database reduction [6,38]. Both `used` and `lbd` can be altered via clause *strengthening* [6] in SUB.

The signature (`sig`) of a clause is computed by hashing its literals to a 32-bit value [16]. It is used to quickly compare clauses. The first literal in a clause is preallocated and stored in the fixed array `literals[1]`. As has been done for the MiniSat solver, we adapted the `union` structure to allow dynamically expanding the `literals` array. This is accepted by NVIDIA's compiler (NVCC). In our previous work [34], we stored a pointer in each clause referencing the first literal, with the literals being in a separate array. This consumes 8 bytes of the clause space. However, `SCLAUSE` only needs 4 bytes for the `literals` array, resulting in the clause occupying 20 bytes in total, including the extra information of the learnt clause, compared to 24 bytes in our previous work.

As implemented in MiniSat, we use the `clauses` field in `CNF` (Fig. 1b) to store the raw bytes of `SCLAUSE` instances with any extra literals in 4-byte buckets with 64-bit reference support. The `cap` variable indicates the total memory capacity available for the storage of clauses, and `size` reflects the current size of the list of clauses. We always have `size` $\leq$ `cap`. The `references` field is used to directly access the clauses by saving for each clause a reference to their first bucket. The mechanism for storing references works in the same way as for clauses.

In addition, in a similar way, an *occurrence table* structure, denoted by `OT`, is created which has a raw pointer to store the 64-bit clause references for each literal in the formula and a member structure `OL`. The creation of an `OL` instance is done in parallel on the GPU for each literal using atomic instructions. For each clause $C$, a thread is launched to insert the occurrences of $C$'s literals in the associated lists.

Initially, we pre-allocate unified memory for `clauses` and `references` which is in size twice as large as the input formula, to guarantee enough space for the original and learnt clauses. This amount is guaranteed to be enough as we enforce that the number of resolvents never exceeds the number of `ORIGINAL` clauses. The `OT` memory is reallocated dynamically if needed after each variable elimination. Furthermore, we check the amount of free available GPU memory before allocation is done. If no memory is available, the inprocessing step is skipped and the solving continues on the CPU.

### 4 Parallel Garbage Collection

Modern sequential SAT solvers implement a *garbage collection* (GC) algorithm to reduce memory consumption and maintain data locality [2, 6, 17].

Since GPU global memory is a scarce resource and coalesced accesses are essential to hide the latency of global memory (see Section 2), we decided to develop an efficient and parallel GC algorithm for the GPU without adding overhead to the GPU computations.

Fig. 2 demonstrates the proposed approach for a simple SAT formula $\mathcal{S} = \{\{a, \bar{b}, c\}, \{a, b, \bar{c}\}, \{d, \bar{b}\}, \{\bar{d}, b\}\}$, in which $\{a, b, \bar{c}\}$ is to be deleted. The figure shows, in addition, how the `references` and `clauses` lists in Fig. 1b are updated for the given formula. The reference for each clause $C$ is calculated based on the sum of the sizes (in buckets) of all clauses preceding $C$ in the list of clauses. For example, the first clause ($C_1$) requires $\alpha + (k - 1) = 5 + 2 = 7$ buckets, where the constant $\alpha$ is the number of buckets needed to store `SCLAUSE`, in our case 20 bytes / 4 bytes, and $k$ is the clause size in terms of the number of literals. Given the number of buckets needed for $C_1$, the next clause ($C_2$) must be stored starting from position 7 in the list of clauses. This position plus the size of $C_2$ determines in a similar way the starting position for $C_3$, and so on.

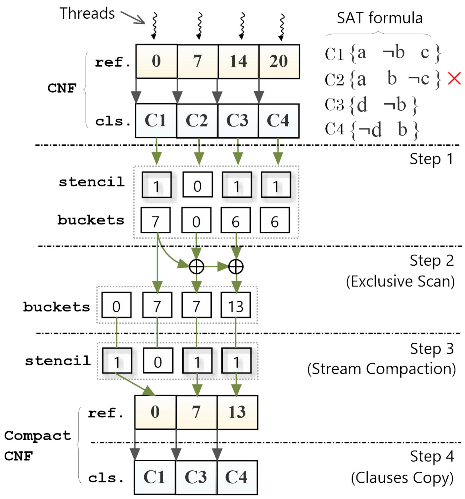

Fig. 2: An example of parallel GC on a GPU

The first step towards compacting the `CNF` instance when $C_2$ is to be deleted is to compute a *stencil* and a list of corresponding clause sizes in terms of numbers of buckets. In this step, each clause $C_i$ is inspected by a different thread that writes a '0'

**Algorithm 1:** Parallel Garbage Collection

```
Input  : __global__ S_in, stencil, buckets, __constant__ α , __shared__ shCls, shLits
Output: numCls, numLits
numCls, numLits ← COUNTSURVIVED(S_in);
S_out ← ALLOCATE(numCls, numLits);
stencil, buckets ← COMPUTESTENCIL(S_in);
buckets ← EXCLUSIVESCAN(buckets);
references(S_out) ← COMPACTREFS(buckets, stencil);
COPYCLAUSES(S_out, S_in, buckets, stencil);
kernel COUNTSURVIVED (S_in):
    register rCls ← 0, rLits ← 0;
    for all i ∈ ⟦ 0, |S_in| ⟧ in parallel
        register C ← S_in[i];
        if state(C) ≠ DELETED then
            rCls ← rCls + 1, rLits ← rLits + |C|;
    if tid < |S_in| then
        shCls[tid] = rCls, shLits[tid] = rLits;
    else
        shCls[tid] = 0, shLits[tid] = 0;
    SYNCTHREADS( );
    for b : blockDim/2, b/2 → 1 do   // b will be blockDim/2, (blockDim/2)/2, ..., 1
        if tid < b then
            shCls[tid] ← shCls[tid] + shCls[tid + b], shLits[tid] ← shLits[tid] + shLits[tid + b];
        SYNCTHREADS( );
    if tid = 0 then
        ATOMICADD(numCls, shCls[tid]), ATOMICADD(numLits, shLits[tid]);
kernel COMPUTESTENCIL (S_in):
    for all i ∈ ⟦ 0, |S_in| ⟧ in parallel
        register C ← S_in[i];
        if state(C) = DELETED then
            stencil[i] ← 0 , buckets[i] ← 0;
        else
            stencil[i] ← 1 , buckets[i] ← α + (|C| − 1);
kernel COPYCLAUSES (S_out, S_in, buckets, stencil):
    for all i ∈ ⟦ 0, |S_in| ⟧ in parallel
        if stencil[i] then
            register & C_dest ← (SCLAUSE &)(clauses(S_out) + buckets[i]);
            C_dest ← S_in[i];
```

at position $i$ of a list named `stencil` if the clause must be deleted, and a '1' otherwise. The size of `stencil` is equal to the number of clauses. In a list of the same size called `buckets`, the thread writes at position $i$ '0' if the clause will be deleted, and otherwise the size of the clause in terms of the number of buckets.

At step 2, a parallel *exclusive-segmented scan* operation is applied on the `buckets` array to compute the new references. In this scan, the value stored at position $i$, masked by the corresponding `stencil`, is the sum of the values stored at positions 0 up to, but not including, $i$. An optimised GPU implementation of this operation is available via the CUDA CUB library [29], which transforms a list of size $n$ in $\log(n)$ iterations. In the example, this results in $C_3$ being assigned reference 7, thereby replacing $C_2$.

At step 3, the `stencil` list is used to update `references` in parallel, which are be kept together in consecutive positions. The standard `DeviceSelect::Flagged` function of the CUB library can be used for this, which uses stream compaction [10]. Finally, the actual clauses are copied to their new locations in `clauses`.

Alg. 1 describes in detail the GPU implementation of the parallel GC. As input, Alg. 1 requires a SAT formula $\mathcal{S}_{in}$ as an instance of `CNF`. The constant $\alpha$ is kept in

GPU constant memory for fast access. The highlighted lines in grey are executed on GPU. To begin GC, we count the number of clauses and literals in the $\mathcal{S}_{in}$ formula after simplification has been applied (line 1). The counting is done via the parallel reduction kernel COUNTSURVIVED, listed at lines 7-23. In kernels, we use two conventions. First of all, with *tid*, we refer to the *block-local* ID of the executing thread. By using this ID, we can achieve that different threads in the same block work on different data, as for instance at lines 13-16. Second of all, we use so-called *grid-stride loops* to process data elements in parallel. An example of this starts at line 9. The statement **for all** $i \in [\![0, N]\!]$ **in parallel** expresses that all natural numbers in the range $[0, N)$ must be considered in the loop, and that this is done in parallel by having each executing thread start with element *tid*, i.e., $i = tid$, and before starting each additional iteration through the loop, the thread adds to $i$ the total number of threads on the GPU. If the updated $i$ is smaller than $N$, the next iteration is performed with this updated $i$. Otherwise, the thread exits the loop. A grid-stride loop ensures that when the range of numbers to consider is larger than the number of threads, all numbers are still processed.

The values *rCls* and *rLits* at line 8 will hold the current number of clauses and literals, respectively, counted by the executing thread. The **register** keyword indicates that the variables are stored in the thread-local register memory. Within the loop at lines 9-12, the counters *rCls*, *rLits* are updated incrementally if the clause at position $i$ in `clauses` is not deleted. Once a thread has checked all its assigned clauses, it stores the counter values in the (block-local) shared memory arrays (*shCls*, *shLits*) at lines 13-14.

A non-participating thread simply writes zeros (line 16). Next, all threads in the block are synchronised by the SYNCTHREADS call. The loop at lines 18-21 performs the actual parallel reduction to accumulate the number of non-deleted clauses and literals in shared memory within thread blocks. In the **for** loop, $b$ is initially set to the number of threads in the block (*blockDim*), and in each iteration, this value is divided by 2 until it is equal to 1 (note that blocks always consist of a power of two number of threads).

The total number of clauses and threads is in the end stored by thread 0, and this thread adds those numbers using atomic instructions to the globally stored counters *numCls* and *numLits* at line 23, resulting in the final output. In the procedure described here, we prevent having each thread perform atomic instructions on the global memory, by which we avoid a potential performance bottleneck. The computed numbers are used to allocate enough memory for the output formula at line 2 on the CPU side.

The kernel COMPUTESTENCIL, called at line 3, is responsible for checking clause states and computing the number of buckets for each clause. The COMPUTESTENCIL kernel is given at lines 24-30. If a clause $C$ is set to `DELETED` (line 27), the corresponding entries in `stencil` and `buckets` are cleared at line 28, otherwise the `stencil` entry is set to 1 and the `buckets` entry is updated with the number of clause buckets.

The EXCLUSIVESCAN routine at line 4 calculates the new references to store the remaining clauses based on the collected buckets. For that, we use the exclusive scan method offered by the CUB library. The COMPACTREFS routine called at line 5 groups the *valid* references, i.e., those flagged by `stencil`, into consecutive values and stores them in `references`($\mathcal{S}_{out}$), which refers to the `references` field of the output formula $\mathcal{S}_{out}$. Finally, copying clause contents (literals, state, etc.) is done in the COPYCLAUSES kernel, called at line 6. This kernel is described at lines 31-35. If a clause in

$\mathcal{S}_{in}$ is flagged by `stencil` via thread $i$, then a new `SCLAUSE` reference is created in `clauses`($\mathcal{S}_{out}$), which refers to the `clauses` field in $\mathcal{S}_{out}$, offset by `buckets`[$i$].

The GC mechanism described above resulted from experimenting with several less efficient mechanisms first. In the first attempt, two atomic additions per thread were performed for each clause, one to move the non-deleted clause buckets and the other for moving the corresponding reference. However, the excessive use of atomics resulted in a performance bottleneck and produced a different simplified formula on each run, that is, the order in which the new clauses were stored depended on the outcome of the atomic instructions. The second attempt was to maintain stability by moving the GC to the host side. However, accessing unified memory on the host side results in a performance penalty, as it implicitly results in copying data to the host side.

### 5 Parallel Inprocessing Procedure

To exploit parallelism in simplifications, each elimination method is applied on multiple variables simultaneously. Doing so is non-trivial, since variables may *depend* on each other; two variables $x$ and $y$ are dependent iff there exists a clause $C$ with $(x \in C \vee \bar{x} \in C) \wedge (y \in C \vee \bar{y} \in C)$. If both $x$ and $y$ were to be processed for simplification, two threads might manipulate $C$ at the same time. To guarantee soundness of the parallel simplifications, we apply our *least constrained variable elections* algorithm (LCVE) [34] prior to simplification. It is responsible for electing a set of mutually independent variables (candidates) from a set of authorised candidates. The remaining variables relying on the elected ones are frozen. These notions are defined by Defs. 1-4.

**Definition 1 (Authorised candidates).** *Given a CNF formula $\mathcal{S}$, we call $\mathcal{A}$ the set of* authorised candidates*: $\mathcal{A} = \{x \mid 1 \leq h[x] \leq \mu \vee 1 \leq h[\bar{x}] \leq \mu\}$, where*
- *$h$ is a histogram array ($h[x]$ is the number of occurrences of $x$ in $\mathcal{S}$).*
- *$\mu$ denotes a given maximum number of occurrences allowed for both $x$ and its negation $\bar{x}$, representing the cut-off point for the LCVE algorithm.*

**Definition 2 (Candidate Dependency Relation).** *We call a relation $\mathcal{D} : \mathcal{A} \times \mathcal{A}$ a* candidate dependency relation *iff $\forall x, y \in \mathcal{A}$, $x\,\mathcal{D}\,y$ implies that $\exists C \in \mathcal{S}.(x \in C \vee \bar{x} \in C) \wedge (y \in C \vee \bar{y} \in C)$*

**Definition 3 (Elected candidates).** *Given a set of authorised candidates $\mathcal{A}$, we call a set $\varphi \subseteq \mathcal{A}$ a set of* elected candidates *iff $\forall x, y \in \varphi.\ \neg(x\,\mathcal{D}\,y)$*

**Definition 4 (Frozen candidates).** *Given the sets $\mathcal{A}$ and $\varphi$, the set of* frozen candidates *$\mathcal{F} \subseteq \mathcal{A}$ is defined as $\mathcal{F} = \{x \mid x \in \mathcal{A} \wedge \exists y \in \varphi.\ x\,\mathcal{D}\,y\}$*

A top-level description of GPU parallel inprocessing is shown in Alg. 2. The blue-colored lines highlight new contributions of the current work compared to our preprocessing algorithm presented in [34]. As input, it takes the current formula $\mathcal{S}_h$ from the solver (executed on the host) and copies it to the device global memory as $\mathcal{S}_d$ (line 1).

Initially, before simplification, we compute the clause signatures and order variables via concurrent streams at lines 2-3. A stream is a sequence of instructions that are executed in issue-order on the GPU [31]. The use of concurrent streams allows the running

**Algorithm 2:** Parallel Inprocessing

**Input** : $\mathcal{S}_h$, $\mu$, *phases*

1 $\mathcal{S}_d \leftarrow$ COPYTODEVICE ($\mathcal{S}_h$);
2 CALCSIGNATURES ($\mathcal{S}_d$, *stream0*);
3 $\mathcal{A} \leftarrow$ ORDERVARIABLES ($\mathcal{S}_d$, *stream1*);
4 **while** $p : 0 \rightarrow$ *phases* **do**
5     SYNCALL ( ) ;    // Synchronize all streams
6     $\mathcal{T} \leftarrow$ CREATEOT ($\mathcal{S}_d$);
7     PROPAGATE ($\mathcal{U}_h$, $\mathcal{S}_d$, $\mathcal{T}$);
8     $\varphi \leftarrow$ LCVE ($\mathcal{S}_d$, $\mathcal{T}$, $\mathcal{A}$, $\mu$);
9     **if** $p =$ *phases* **then**
10         ERE ($\mathcal{S}_d$, $\mathcal{T}$, $\varphi$);
11         break;
12     SORTOT ($\mathcal{T}$, $\varphi$, LISTKEY);
13     $\mathcal{U}_d \leftarrow$ ELIMINATE ($\mathcal{S}_d$, $\mathcal{T}$, $\varphi$) ;    // Applies VE, SUB, and BCE
14     $\mathcal{U}_h \leftarrow$ COPYTOHOSTASYNC ($\mathcal{U}_d$, *stream1*);
15     COLLECT ($\mathcal{S}_d$, *stream2*);
16     $\mu \leftarrow \mu \times 2$;
17 **device function** LISTKEY *(a, b)***:**
18     $C_a \leftarrow \mathcal{S}_d[a], C_a \leftarrow \mathcal{S}_d[b]$ ;    // $C_a = \{x_1, x_2, \ldots, x_k\}, C_b = \{y_1, y_2, \ldots, y_k\}$
19     **if** $|C_a| \neq |C_b|$ **then return** $C_a < C_b$ ;
20     **if** $x_1 \neq y_1$ **then return** $x_1 < y_1$ ;
21     **if** $x_2 \neq y_2$ **then return** $x_2 < y_2$ ;
22     **if** $|C_a| > 2 \wedge (x_k \neq y_k)$ **then return** $x_k < y_k$ ;
23     **else return** $sig(C_a) < sig(C_b)$ ; of multiple GPU kernels concurrently, if there are enough resources. The ORDERVARIABLES routine produces an ordered array of authorised candidates $\mathcal{A}$ following Def. 1. The **while** loop at lines 4-16 applies VE, SUB, and BCE, for a configured number of iterations (indicated by *phases*), with increasingly large values of the threshold $\mu$. Increasing $\mu$ exponentially allows LCVE to elect additional variables in the next elimination phase since after a phase is executed on the GPU, many elected variables are eliminated. The ERE method is computationally expensive. Therefore, it is only executed once in the final iteration, at line 10. At line 5, SYNCALL is called to synchronize all streams being executed. At line 6, the occurrence table $\mathcal{T}$ is created. The LCVE routine produces on the host side an array of elected mutually independent variables $\varphi$, in line with Def. 3.

The parallel creation of the occurrence lists in $\mathcal{T}$ results in the order of these lists being chosen non-deterministically. This results in the ELIMINATE procedure called at line 13, which performs the parallel simplifications, to produce results non-deterministically as well. To remedy this effect, the lists in $\mathcal{T}$ are sorted according to a unique key in ascending order. Besides the benefit of stability, this allows SUB to abort early when performing subsumption checks. The sorting key function is given as the device function LISTKEY at lines 17-24. It takes two references $a, b$ and fetches the corresponding clauses $C_a, C_b$ from $\mathcal{S}_d$ (line 18). First, clause sizes are tested at line 19. If they are equal, the first, the second, and the last literal in each clause are checked, respectively, at lines 20-22. Otherwise, clause signatures are tested at line 23. CADICAL implements a similar function, but only considers clause sizes [6]. The SORTOT routine launches a kernel to sort the lists pointed to by the variables in $\varphi$ in parallel. Each thread runs an insertion sort to in-place swap clause references using LISTKEY.

The ELIMINATE procedure at line 13 calls SUB to remove any subsumed clauses or strengthen clauses if possible, after which VE is applied, followed by BCE. The SUB and BCE methods call kernels that scan the occurrence lists of all variables in $\varphi$ in parallel. For more information on this, see [34]. The VE method uses a new parallel approach, which is explained in Section 6. Both the VE and SUB methods may add new unit clauses atomically to a separate array $\mathcal{U}_d$. The propagation of these units cannot be done immediately on the GPU due to possible data races, as multiple variables in a clause may occur in unit clauses. For instance, if we have unit clauses $\{a\}$ and $\{b\}$, and these would be processed by different threads, then a clause $\{\bar{a}, \bar{b}, c\}$ could be updated by both threads simultaneously. Thus, this propagation is delayed until the next iteration, and performed by the host at line 7. Note that $\mathcal{T}$ must be recreated first to consider all resolvents added by VE during the previous phase. The ERE method at line 10 is executed only once at the last phase (*phases*) before the loop is terminated. Section 7 explains in detail how ERE can be effective in simplifying both `ORIGINAL` and `LEARNT` clauses in parallel. At line 14, new units are copied from the device to the host array $\mathcal{U}_h$ asynchronously via *stream1*. The COLLECT procedure does the GC as described by Alg. 4 via *stream2*. Both streams are synchronized at line 5.

### 6 Three-Phase Parallel Variable Elimination

The BVIPE algorithm in our previous work [34] had a main shortcoming due to the heavy use of atomic operations to add new resolvents. Per eliminated variable, two atomic instructions were performed, one for adding new clauses and the other for adding new literals. Besides performance degradation, this also resulted in the order of added clauses being chosen non-deterministically, which impacted reproducibility (even though the produced formula would always at least be logically the same).

The approach to avoiding the excessive use of atomic instructions when adding new resolvents is to perform parallel VE in *three phases*. The first phase scans the constructed list $\varphi$ to identify the elimination type (e.g., resolution or gate substitution) of each variable and to calculate the number of resolvents and their corresponding buckets.

The second phase computes an exclusive scan to determine the new references for adding resolvents, as is done in our GC mechanism (Section 4). At the last phase, we store the actual resolvents in their new locations in the simplified formula. For solution reconstruction, we use an atomic addition to count the resolved literals. The order in which they are resolved is irrelevant. The same is done for adding units. For the latter, experiments show that the number of added units is relatively small compared to the eliminated variables, hence the penalty of using atomic instructions is almost negligible. It would be overkill to use a segmented scan for adding literals or units.

At line 1 of Alg. 3, phase 1 is executed by the VARIABLESWEEP kernel (given at lines 15-27). Every thread scans the clause set of its designated literals $x$ and $\bar{x}$ (line 17). References to these clauses are stored at $\mathcal{T}_x$ and $\mathcal{T}_{\bar{x}}$. Moreover, register variables $t, \beta, \gamma$ are created to hold the current *type*, number of *added clauses*, and number of *added literals* of $x$, respectively. If $x$ is *pure* at line 19, then there are no resolvents to add and the clause sets of $x$ and $\bar{x}$ are directly marked as `DELETED` by the routine TOBLIVION. Moreover, this routine adds the marked literals atomically to `resolved`. At line 22, we

**Algorithm 3:** Three-Phase Parallel Variable Elimination

```
Input : __global__ φ, S_d, T, U_d, resolved, type, buckets, added, __constant__ α
1  resolved, type, buckets, added ← VARIABLESWEEP(φ, S_d, T);
2  last_added ← −1, last_idx ← −1, last_cref ← −1, last_C ← ∅;
3  for j : |φ| − 1, j − 1 → 0 do    // find index and # resolvents of last eliminated x
4      if type[j] ≠ 0 then
5          last_idx ← j, last_added ← added[j]; break;
6  buckets ← EXCLUSIVESCAN (buckets, SIZE(clauses), stream0);
7  added ← EXCLUSIVESCAN (added, SIZE(references), stream1);
8  SYNCALL( );
9  numCls ← last_added + added[last_idx];
10 last_cref ← references[numCls − 1], last_C ← clauses[last_cref];
11 numBuckets ← last_cref + (α + SIZE(last_C) − 1);
12 RESIZE(clauses, numBuckets), RESIZE(references, numCls);
13 S_d, U_d ← VARIABLERESOLVENT(φ, S_d, T, type, buckets, added);
14
15 kernel VARIABLESWEEP (φ, S_d, T):
16     for all i ∈ ⟦ 0, |φ| ⟧ in parallel
17         register x ← φ[i], T_x ← T[x], T_x̄ ← T[x̄], t ← NONE, β ← 0, γ ← 0;
18         type[i] ← 0, buckets[i] ← 0, added[i] ← 0 ;               // initially reset
19         if T_x = ∅ ∨ T_x̄ = ∅ then                      // check if x is a pure literal
20             resolved ← TOBLIVION(x, S_d, T_x, T_x̄);
21         else
22             t, β, γ ← GATEREASONING (x, S_d, T_x, T_x̄, σ);
23             if t ≠ GATE then
24                 t, β, γ ← MAYRESOLVE (x, S_d, T_x, T_x̄) ;     // t may set to RESOLUTION
25             if t ≠ 0 then                                       // x can be eliminated
26                 type[i] ← t, added[i] ← β, buckets[i] ← α × β + (γ − β);
27                 resolved ← TOBLIVION(x, S_d, T_x, T_x̄);
28 kernel VARIABLERESOLVENT (φ, S_d, T, type, buckets, added):
29     for all i ∈ ⟦ 0, |φ| ⟧ in parallel
30         register x ← φ[i], T_x ← T[x], T_x̄ ← T[x̄];
31         register t ← type[i], cref ← buckets[i], rpos = added[i];
32         if t = RESOLUTION then
33             (S_d, U_d) ← (S_d, U_d) ∪ RESOLVE(x, S_d, T_x, T_x̄, rpos, cref);
34         if t = GATE then
35             (S_d, U_d) ← (S_d, U_d) ∪ SUBSTITUTE(x, S_d, T_x, T_x̄, rpos, cref);
```

check first if $x$ contributes to a logical gate using the routine GATEREASONING, and save the corresponding $\beta$ and $\gamma$. If this is the case, the type $t$ is set to `GATE`, otherwise we try resolution at line 24. The condition $\beta \leq (|\mathcal{T}_x| + |\mathcal{T}_{\bar{x}}|)$ is tested implicitly by MAYRESOLVE to limit the number of resolvents per $x$. If $t$ is set to a nonzero value (line 25), the `type` and `added` arrays are updated correspondingly. The total number of buckets needed to store all added clauses is calculated by the formula $(\alpha \times \beta + (\gamma - \beta))$ and stored in $\texttt{buckets}[i]$ at line 26. After `type` and `added` have been completely constructed, the loop at lines 3-4 identifies the index of the last variable eliminated starting from position $|\varphi| - 1$. If the condition at line 4 holds, index $j$ and the number of underlying resolvents are saved to $last_{idx}$ and $last_{added}$, respectively. These values will be used later to set the new size of the simplified formula $\mathcal{S}_d$ on the host side.

Phase 2 is now ready to apply EXCLUSIVESCAN on the `added` and `buckets` lists. Both `clauses` and `references` refer to the structural members of $\mathcal{S}_d$, as described in Fig. 1b. The procedure at line 6 takes the old size of `clauses` to offset the calculated references of the added resolvents. The SIZE routine returns the size of the input structure. Similarly, the second call at line 7 takes the old size of `references` and calculates the new indices for storing new references. Both scans are executed concurrently

**Algorithm 4:** Parallel Eager Redundancy Elimination for Inprocessing

```
Input : __global__ φ, S_d, T
1  kernel ERE (φ, S_d, T):
2      for all i ∈ ⟦ 0, |φ| ⟧^y in parallel
3          x ← φ[i];
4          for C ∈ S_d[T[x]] do
5              for C' ∈ S_d[T[x̄]] do
6                  if (C_m ← RESOLVE (x, C, C')) ≠ ∅ then
7                      if state(C) = LEARNT ∨ state(C') = LEARNT then
8                          st ← LEARNT
9                      else
10                         st ← ORIGINAL
11                     FORWARDEQUALITY (C_m, S_d, T, st);
12 device function FORWARDEQUALITY (C_m, S_d, T, st):
13     minList ← FINDMINLIST (T, C_m);
14     for all i ∈ ⟦ 0, |minList| ⟧^x in parallel
15         C ← S_d[minList[i]];
16         if C = C_m ∧ (state(C) = LEARNT ∨ state(C) = st) then state(C) ← DELETED ;
```

via *stream0* and *stream1*, and are synchronized by the SYNCALL call at line 8. After the exclusive scan, the last element in `added` gives the total number of clauses in $\mathcal{S}_d$ minus the resolvents added by the last eliminated variable. Therefore, adding this value to $last_{added}$ gives the total number of clauses in $\mathcal{S}_d$ (line 9). At line 10, the last clause $last_C$ and its reference $last_{cref}$ are fetched. At line 11, the number of buckets of $last_C$ is added to $last_{cref}$ to get the total number of buckets *numBuckets*. The *numBuckets* and *numCls* are used to resize `clauses` and `references`, respectively, at line 12.

Finally, in phase 3, we use the calculated indices in `added` and `buckets` to guide the new resolvents to their locations in $\mathcal{S}_d$. The kernel is described at lines 28-35. Each thread either calls the procedure RESOLVE or SUBSTITUTE, based on the type stored for the designated variables. Any produced units are saved into $\mathcal{U}_d$ atomically. The *cref* and *rpos* variables indicate where resolvents should be stored in $\mathcal{S}_d$ per variable $x$.

### 7 Eager Redundancy Elimination

Alg. 4 describes a *two-dimensional* kernel, in which from each thread ID, an $x$ and $y$ coordinate is derived. This allows us to use two nested grid-stride loops. In the loops, we specify which of the two coordinates should be used to initialise $i$ in the first iteration.

Based on the kernel's *y-dimension* ID (line 2), each thread merges where possible two clauses of its designated variable $x$ and its complement $\bar{x}$ (lines 3-6), and writes the result in shared memory as $C_m$. This new clause is produced by the routine RESOLVE at line 6. At lines 7-10, we check if one of the resolved clauses is `LEARNT`, and if so, the state *st* of $C_m$ is set to `LEARNT` as well, otherwise it is set to `ORIGINAL`. This state of $C_m$ will guide the FORWARDEQUALITY routine called at line 11 to search for redundant clauses of the same type. This routine is a device function, as it can only be called from a kernel, and is described at lines 12-17. In this function, the $x$-dimension of the thread ID is used to search the clauses referenced by the minimum occurrence list *minList*, which is produced by FINDMINLIST at line 13. It has the minimum size among the lists of all literals in $C_m$. If a clause $C$ is found that is equal to $C_m$ and is either `LEARNT` or has a state equal to the one of $C_m$, it is set to `DELETED` (lines 16).

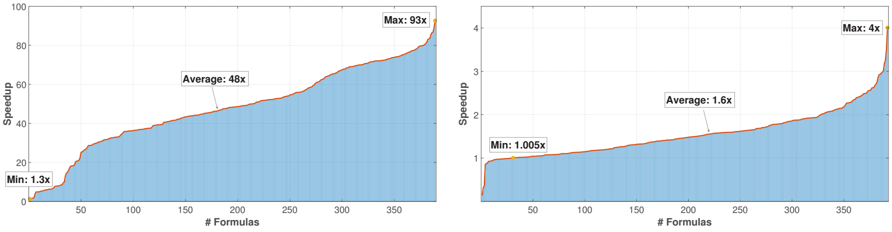

(a) Parallel GC vs. sequential (b) Three-Phase VE vs. atomic version

Fig. 3: Speedup of the proposed VE and GC algorithms on the benchmark suite

## 8 Experiments

We implemented the proposed algorithms in PFROST-GPU[3] with CUDA C++ version 11.0 [31]. We evaluated all GPU experiments on an NVIDIA Titan RTX GPU. This GPU has 72 SMs (64 cores each), 24 GB global memory and 48 KB shared memory. The GPU operates at a base clock of 1.3 GHz (boost: 1.7 GHz). The GPU machine was running Linux Mint v20 with an Intel Core i5-7600 CPU of 3.5 GHz base clock speed (turbo: 4.1 GHz) and a system memory of 32 GB.

We selected 493 SAT problems from the 2013-2020 SAT competitions. All formulas larger than 5 MB in size are chosen, excluding redundancies (repeated CNFs across competitions). For very small problems, the GPU is not really needed, as only few variables and clauses can be removed. The selected problems encode around 70+ different real-world applications, with various logical properties.

In the experiments, besides the implementations of our new GPU algorithms, we involved a CPU-only version of PARAFROST (PFROST-CPU), and the CADICAL [6] SAT solver for the solving of problems, and executed these on the compute nodes of the Lisa CPU cluster[4]. Each problem was analysed in isolation on a separate computing node. Each computing node had an Intel Xeon Gold 6130 CPU running at a base clock speed of 2.1 (turbo: 3.7) GHz with 96 GB of system memory, and runs on Debian Linux operating system. With this information, we adhere to all five principles laid out in the SAT manifesto (version 1) [9], noting that we also included problems older than three years, to have a sufficient number of large problems to work with.

**SAT-Simplification Speedup.** Figure 3 discusses the performance evaluation of the GPU Algorithms 1 and 3 compared to their previous implementations in SIGMA [34]. For these experiments, we set $\mu$ and *phases* initially to 32 and 5, respectively. Preprocessing is only enabled to measure the speedup. Fig. 3a shows the speedup of running parallel GC against a sequential version on the host. Clearly, for almost all cases, Alg. 1 achieved a drastic acceleration when executed on the device with a maximum speed up of 93$\times$ and an average of 48$\times$. Fig. 3b reveals how fast the 3-phase parallel VE is

[3] Solvers/formulas are available at https://gears.win.tue.nl/software/parafrost.

[4] This work was carried out on the Dutch national e-infrastructure with the support of SURF Cooperative.

compared to version using more atomic instructions. On average, the new algorithm is twice as fast as the old BVIPE algorithm [34]. In addition, we get reproducible results.

**SAT-Solving.** These experiments provide a thorough assessment of our CPU/GPU solver, the CPU-only version, and CaDiCaL on SAT solving with preprocessing + inprocessing turned on. The features *walksat*, *vivification* and *probing* [6] are disabled in CaDiCaL as they are not yet supported in ParaFROST. As in ParaFROST, all elimination methods in CaDiCaL are turned on with a bound on the occurrence list size set to 30,000. The same parameters for the search heuristics are used for all experiments. However, we delay the scheduling of inprocessing in ParaFROST until 4,000 of the fixed (root) variables are removed. The occurrence limit $\mu$ is bounded by 32 in CaDiCaL. On the other hand, we start with 32 and double this value every new *phase* as shown in Alg. 2. These extensions increase the likelihood of doing more work on the GPU. The timeout for all experiments is set to 5,000 seconds. The timeout for the sequential solvers has a 6% tolerance (i.e., is 5,300 seconds in total) to compensate for the different CPU frequencies of the GPU machine and the cluster nodes.

Figure 4 demonstrates the runtime results for all solvers over the benchmark suite. Subplot (a) shows the total time (simplify + solving) for all formulas. Data are sorted w.r.t. the $x$-axis. The simplify time accounts data transfers in PFROST-GPU. Overall, PFROST-GPU dominates over PFROST-CPU and CaDiCaL. Subplot (b) demonstrates the solving impact of PFROST-GPU versus CaDiCaL on `SAT/UNSAT` formulas. PFROST-GPU seems more effective on `UNSAT` formulas than CaDiCaL. Collectively, PFROST-GPU performed faster on 196 instances (58% out of all solved), in which 18 formulas were unsolved by CaDiCaL.

Subplots (c) and (d) show simplification time and its percentage of the total processing time, respectively. Clearly, the CPU/GPU solver outperforms its sequential counterpart due to the parallel acceleration. Plot (d) tells us that PFROST-GPU keeps the workload in the region between 0 and 20% as the elimination methods are scheduled on a bulk of mutually independent variables in parallel. In CaDiCaL, variables and clauses are simplified sequentially, which takes more time. Plot (e) shows the effectiveness of ERE on formulas with successful clause reductions. The last plot (f) reflects the overall efficiency of parallel inprocessing on variables and clauses (learnt clauses are included). Data are sorted in descending order. Reductions can remove up to 90% and 80% of the variables and clauses, respectively.

### 9 Related Work

A simple GC monitor for GPU term rewriting has been proposed by van Eerd *et al.* [18]. The monitor tracks deleted terms and stores their indices in a list. New terms can be added at those indices. The authors in [1, 26] investigated the challenges for offloading garbage collectors to an Accelerated Processing Unit (APU). Matthias *et al.* [39] introduced a promising alternative for stream compaction [10] via parallel defragmentation on GPUs. Our GC, on the other hand, is tailored to SAT solving, which allows it to be simple yet efficient. Regarding inprocessing, Järvisalo *et al.* [23] introduced certain rules to determine how and when inprocessing techniques can be applied. Acceleration of the DPLL SAT solving algorithm on a GPU has been done in [15], where

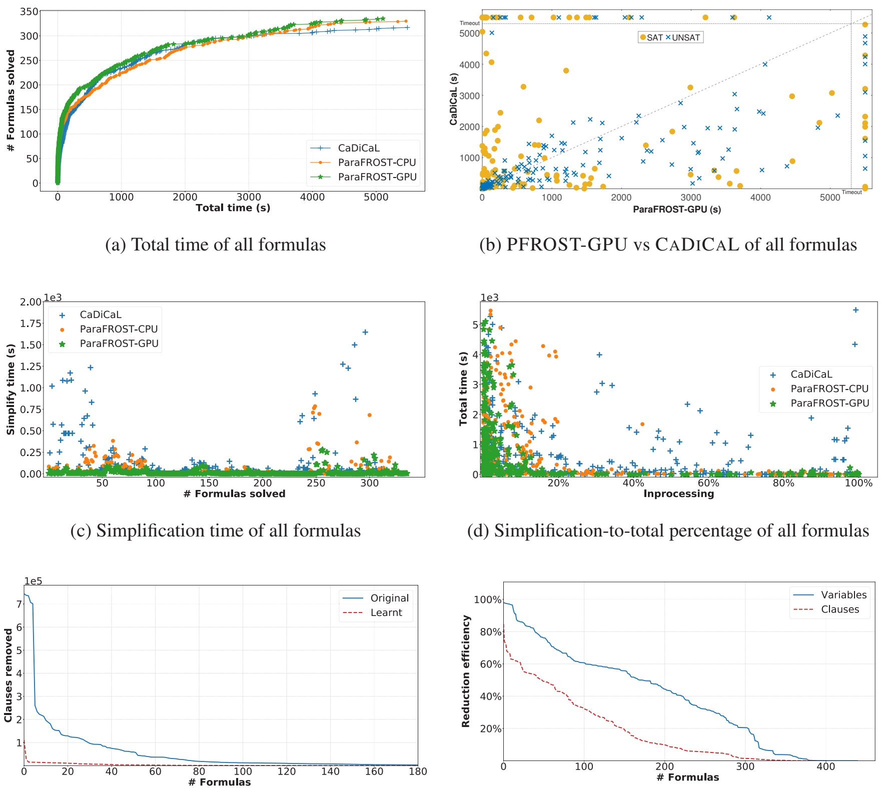

(a) Total time of all formulas

(b) PFROST-GPU vs CADICAL of all formulas

(c) Simplification time of all formulas

(d) Simplification-to-total percentage of all formulas

(e) ERE reductions

(f) Inprocessing reduction efficiency

Fig. 4: SAT Solving Statistics some parts of the search were performed on a GPU and the remainder is handled by the CPU. Incomplete approaches are more amenable to be executed entirely on a GPU, e.g., an approach using metaheuristic algorithms [44]. We are the first to work on GPU inprocessing in modern CDCL solvers.

### 10 Conclusion

We have shown that GPU-accelerated inprocessing significantly reduces simplification time in SAT solving, allowing more problems to be solved. Parallel ERE and VE can be performed efficiently on many-core systems, producing impactful reductions on both original and learnt clauses in a fraction of a second, even for large problems. The proposed parallel GC achieves a substantial speedup in compacting SAT formulas on a GPU, while stimulating coalesced accessing of clauses.

Concerning future work, the results suggest to continue taking the capabilities of GPU inprocessing further by supporting more simplification techniques.
